# Differences in Cortical Representation and Structural Connectivity of Hands and Feet between Professional Handball Players and Ballet Dancers

**DOI:** 10.1155/2016/6817397

**Published:** 2016-05-09

**Authors:** Jessica Meier, Marlene Sofie Topka, Jürgen Hänggi

**Affiliations:** Division Neuropsychology, Department of Psychology, University of Zurich, 8050 Zurich, Switzerland

## Abstract

It is known that intensive training and expertise are associated with functional and structural neuroadaptations. Most studies, however, compared experts with nonexperts; hence it is, specifically for sports, unclear whether the neuroplastic adaptations reported are sport-specific or sport-general. Here we aimed at investigating sport-specific adaptations in professional handball players and ballet dancers by focusing on the primary motor and somatosensory grey matter (GM) representation of hands and feet using voxel-based morphometry as well as on fractional anisotropy (FA) of the corticospinal tract by means of diffusion tensor imaging-based fibre tractography. As predicted, GM volume was increased in hand areas of handball players, whereas ballet dancers showed increased GM volume in foot areas. Compared to handball players, ballet dancers showed decreased FA in both fibres connecting the foot and hand areas, but they showed lower FA in fibres connecting the foot compared to their hand areas, whereas handball players showed lower FA in fibres connecting the hand compared to their foot areas. Our results suggest that structural adaptations are sport-specific and are manifested in brain regions associated with the neural processing of sport-specific skills. We believe this enriches the plasticity research in general and extends our knowledge of sport expertise in particular.

## 1. Introduction

Numerous studies have shown that the human brain changes its structure and function as a consequence of training, expertise, and environmental influences throughout the entire lifespan [1–4]. These structural and functional adaptations, called neuroplasticity, can be measured in vivo by using a variety of magnetic resonance imaging (MRI) techniques [5]. Among the first studies that investigated practice-dependent structural neuroplasticity by using MRI were the different juggling studies conducted by Draganski and colleagues [6–9]. One of the main results of these studies is a bilateral expansion in grey matter (GM) in the midtemporal area (hMT/V5) as a consequence of a three-month juggling training [7], suggesting that even short-term practice of a specific task is associated with structural adaptations in relevant brain regions. In addition, these longitudinal studies also suggest that the structural changes reported are the consequences of the training and are hence evoked by neuroplastic processes rather than just the result of a genetic predisposition for a particular neural trait. Therefore, it is conceivable to assume that long-term training (years to decades) in a specific task evokes structural brain adaptations too and that those adaptations can be measured using structural MRI in cross-sectional study designs, in which experts of one domain are compared with nonexperts. Indeed, there is strong evidence from cross-sectional structural MRI studies that sensory, motor, and cognitive training modulates brain morphology [3, 5, 10–12]. However, the number of structural imaging studies in which professional athletes were compared to nonathletes with respect to differences in GM and white matter (WM) are assessable (for an overview see Table 1 of [13]). In athletes compared with controls, these studies revealed mainly increase but also decrease in GM and WM volume in primary motor (M1) and somatosensory (S1) cortex, supplementary motor area (SMA), premotor cortex, basal ganglia, thalamus, and cerebellum as well as differences in fractional anisotropy (FA) of the corticospinal tract (CST). Such structural adaptations have been observed, for example, for basketball players [14], judo wrestlers [15], endurance athletes and martial artists [16, 17], golf players [18], ballet dancers [19], and handball players [13]. However, even if these findings indicate that long-term physical training changes both GM and WM and the fact that particularly those brain regions are shown to be modulated which are assumed to be specifically associated with the skills of the trained activity, it remains unknown whether these neuroplastic adaptations are indeed sport-specific or just sport-general.

To the best of our knowledge there are only three previous studies comparing two different groups of athletes. First, this is the work recently published by Chang and colleagues [16] who investigated white matter integrity in the basal ganglia between elite professional athletes specializing in running, martial arts, and control subjects [16]. These authors showed lower FA and higher mean diffusivity (MD) values in the globus pallidus internal segment of both athletic groups compared to the control group but revealed no differences between the two athletic groups. These findings indeed support previous studies which showed that professional sports are associated with structural changes in specific brain regions, but they did not clarify whether different types of sport can induce distinct structural differences in the brain [16]. Second, Schlaffke and colleagues [17] investigated GM volumes again in martial athletes, endurance athletes, and nonathletes. They revealed increased GM volumes in the SMA in both groups of athletes when compared with nonathletes but also revealed brain structures adapted in one sport but not in the other. For example, endurance athletes compared with martial athletes showed increased GM volume in the hippocampus and parahippocampal gyrus, a fact interpreted by the authors to be related to the different metabolic profiles of the two sports; that is, martial sport is rather anaerobic and endurance sport is rather aerobic [17]. The third publication is a study conducted by Wenzel and colleagues [20] who investigated the influence of simple fast foot movements on functional and structural brain alterations between power (sprinters, jumpers, and throwers) and endurance (middle- and long-distance runners) athletes. Behaviourally, a significant difference between the two sports groups was found in movement velocity of plantarflexion. Furthermore, functional magnetic resonance imaging (fMRI) analysis indicated that fast plantarflexions were accompanied by increased activity in the cerebellar anterior lobe but surprisingly without significant differences between the two groups of athletes. The same region, however, indicated increased GM volume for the power athletes compared to the endurance athletes what the authors interpreted as evidence of a unique structural feature in the brains of speed-trained athletes [20]. Based on these studies and the fact that little is known about sport-specific brain plasticity so far, the present study aimed at questioning whether brain structures of two different sports groups show different plastic adaptations.

In two previous studies of our group [13, 19] the structure of brain areas relevant for motor and sensory control was investigated in professional female ballet dancers [19] and in professional female handball players [13], each sport group compared to different control groups. Based on these two studies we compared the same group of ballet dancers with the same group of handball players with respect to their M1 and S1 hand and foot representations by means of the voxel-based morphometry (VBM) technique as well as with respect to their fibre connections via the CST by means of fibre tractography based of diffusion tensor imaging (DTI) data.

Classical ballet dancing is a very demanding and well-defined form of dancing requiring not only high levels of physical strength, balance, and flexibility [21], but also precise positioning in space and synchronization to music [19]. Ballet dancing differs from other dance forms with regard to its special body position:* en dehors*. This constant outward rotation of the legs starting in the hip, so that knees and feet are turned outward, forms the basis of ballet. Every sequence of dancing steps must start and end with one of five basic positions (1st, 2nd, 3rd, 4th, or 5th, resp.). Specifications, such as toe dancing (*en pointe*), further increase complexity [22]. In addition to the very demanding technical and physical basis involving mainly legs and feet, the correct position of the hands and facial and emotional expressions and the need for seemingly easiness pose further challenges to a ballet dancer [22].

Contrary to classical ballet, the main focus of handball lies on physical abilities involving both hands. In addition to general physical aspects such as running, jumping, endurance, mobility, speed, and skills, throwing the ball including throwing speed represents major facets of a successful player [23]. The basis is a specific throwing technique, a well-timed movement pattern involving different body parts, mostly arms and hands [23, 24]. Different throws require distinct movement patterns; a common feature, however, is that all of them require precise coordination of upper and lower limbs. The most frequently used technique (*jump shot*), for instance, is comprised of a three-step rhythm (left-right-left) towards the goal, in which a right-handed player jumps off with the left leg, throws the ball with the right hand, and cushions the jump with the right leg before landing again on both feet [25].

Due to the fact that the feet are more important in ballet dancers and the hands are more important in handball players, we expected to find differences in the M1 and S1 representations of the hand and foot indicated by greater GM volume alterations in the hand areas of handball players compared to greater GM volume alterations in the foot areas of ballet dancers. We also predicted differences in the white matter fibres connecting the hand and foot representations via the CST indicated by greater alterations in FA of fibres connecting the foot areas in ballet dancers and of fibres connecting the hand areas in handball players, respectively.

## 2. Materials and Methods

### 2.1. Subjects

We investigated 44 women aged between 18 and 37 years (mean = 23.92 years, standard deviation (SD) = 3.83 years) of whom 10 are professional ballet dancers (mean = 21.94 years, SD = 3.03 years), 12 are professional handball players (mean = 23.25 years, SD = 2.96 years), and 22 are control women (mean = 25.19, SD = 4.20). The control group is composed of the same control participants of the before mentioned two previous studies of our group with 10 control women of the ballet study [19] and 12 control women of the handball study [13]. In order to avoid the impression that the present study is just a redundant follow-up paper of the two studies already published, we would like to emphasize that for the present paper we posed new hypotheses, applied a ROI-based approach, preprocessed the data in a different way, applied entirely new analyses and contrasts, and obtained new findings. There were no significant differences between these two control groups with respect to demographic or global brain measures. Control subjects were matched with respect to handedness and age and were only included if they did not work out any form of sport regularly. Ballet dancers were recruited from different ballet schools in Switzerland, trained ballet dancing for on average 14.2 years (SD = 3.26 years), and started dance training with an average of 7.3 years (SD = 2.50 years). The handball players played in the two highest national leagues, trained handball playing for on average of 12.67 years (SD = 2.67 years), and started handball training with an average of 10.58 years (SD = 2.97 years). None of the participants reported past neurological, neuropsychological, or psychiatric diseases and denied taking drugs and illegal medication. All participants were right handed as assessed with Annett's handedness questionnaires for ballet dancers (and their corresponding control women, resp.) [27] and with the hand dominance test for handball players (and their corresponding control women, resp.) [28, 29]. The fact that the handedness of the participants investigated in the current study was tested with different inventories is due to the fact that the groups come from two different previous studies. However, the different handedness inventories used for the two sports groups are comparable to each other. Further details of these sportswomen and their corresponding control women can be found elsewhere [13, 19].

### 2.2. Magnetic Resonance Imaging Data Acquisition

MRI scans were acquired on a 3.0 Tesla Philips Intera whole body scanner (Philips Medical Systems, Best, Netherlands) equipped with a transmit-receive body coil and a commercial eight-element head coil array which is capable of sensitivity encoding (SENSE).

Handball players and ballet dancers (and their corresponding control participants) had identical T1-weighted images. A volumetric 3D T1-weighted gradient echo sequence (turbo field echo) image was measured with a spatial resolution of 1 × 1 × 1.5 mm^3^ (acquisition matrix 224 × 224 pixels, 90 slices) and reconstructed to a resolution of 0.86 × 0.86 × 0.75 mm^3^ (reconstructed matrix 256 × 256 pixels, 180 slices). It is important to note that this type of MRI signal interpolation does not have an adverse effect on the quality of the data and we do not suggest that our MR images are of a better spatial resolution than the measured one of 1 × 1 × 1.5 mm^3^. Further imaging parameters were field of view FOV = 220 × 220 mm^2^, echo-time TE = 2.3 ms, repetition-time TR = 20 ms, and flip-angle *α* = 20°.

Handball players and ballet dancers (and their corresponding control groups) had slightly different diffusion-weighted sequences. For handball players, a diffusion-weighted spin echo, echo-planar imaging sequence was used to obtain diffusion-weighted scans with a measured spatial resolution of 2.29 × 2.34 × 2.50 mm^3^ (acquisition matrix 96 × 94 pixels, 55 slices) and a reconstructed spatial resolution of 1.72 × 1.72 × 2.5 mm^3^ (reconstructed matrix 128 × 128 pixels, 55 slices). Further imaging parameters were FOV = 220 × 220 mm^2^; TE = 50.0 ms; TR = 11,300 ms; *α* = 90°; SENSE R = 2.1; *b*-value *b* = 1,000 s/mm^2^; and number of averages = 2. Diffusion was measured in 15 noncollinear directions preceded by a non-diffusion-weighted volume (reference volume). For ballet dancers, a diffusion-weighted spin echo, echo-planar imaging sequence was used to obtain diffusion-weighted scans with a measured spatial resolution of 2.08 × 2.13 × 2.00 mm^3^ (acquisition matrix 96 × 96 pixels, 50 slices) and a reconstructed spatial resolution of 1.56 × 1.56 × 2.0 mm^3^ (reconstructed matrix 128 × 128 pixels, 50 slices). Further imaging parameters were FOV = 200 × 200 mm^2^; TE = 50.0 ms; TR = 10,166 ms; *α* = 90°; SENSE R = 2.1; *b*-value *b* = 1,000 s/mm^2^; and number of averages = 2. Diffusion was measured in 15 noncollinear directions preceded by a non-diffusion-weighted volume (reference volume).

### 2.3. Voxel-Based Morphometry

Between-group differences in GM volume were evaluated by using VBM [30, 31]. T1-weighted MRI scans were preprocessed and analysed with the FSL-VBM tool [32] (http://fsl.fmrib.ox.ac.uk/fsl/fslwiki/FSLVBM), an optimised VBM protocol [33] which is implemented in the FMRIB software library (FSL) version 5.0.5 [34] (http://www.fmrib.ox.ac.uk/fsl/). First, structural images were brain-extracted and GM-segmented before being registered to the Montreal neurological institute (MNI) 152 standard space using nonlinear registration [35]. The resulting images were averaged and flipped along the *x*-axis to create a left-right symmetric, study-specific GM template. Second, all native GM images were nonlinearly registered to this study-specific template and “modulated” to correct for local expansion (inflation) and contraction (deflation) due to the nonlinear component of the spatial transformation. The mean values of the regions of interest (ROIs) (see below) were then extracted from the unsmoothed GM volume maps.

### 2.4. Regions of Interest Definition

In the present study we focused on the M1 and S1 representation of the hands and feet as well as on their white matter connections via the CST to the brain stem. Spherical ROIs with a radius of 8 mm were created using the WFU Pickatlas [36, 37] implemented in statistical parametric mapping software version 8 (http://www.fil.ion.ucl.ac.uk/spm/). The coordinates of the eight ROIs (hand/foot, M1/S1, left/right) were drawn from a study that actually investigated the somatotopic organisation of cerebrocerebellar connections in 1000 subjects using fMRI in the resting state, but this study also localized the hand, foot, and tongue representations by using motor-task fMRI in 26 subjects [26]. Due to the fact that the spherical ROIs of FOOT_M1/R_ (*x* = 6, *y* = −26, *z* = 76) and FOOT_S1/R_ (*x* = 10, *y* = −42, *z* = 74) overlap with a radius of 8 mm, we shifted the *x*-coordinate from 6 to 9 mm for M1 and from 10 to 9 for S1. The same shift was applied to the FOOT_M1/L_ (*x* = −6, *y* = −26, and *z* = 76) and FOOT_S1/L_ (*x* = −10, *y* = −42, and *z* = 74); that is, the *x*-coordinate was increased from −6 to −9 for M1 and reduced from −10 to −9 for S1. The Montreal neurological institute (MNI) space coordinates are summarised in Table 1.

The reason why we used the MNI coordinates of the above-mentioned study [26] is that we have not acquired functional localizers and we therefore were dependent on already acquired functional localizers of our predefined ROIs. Since we have not found any fMRI study acquiring functional localizers of both the hand and foot areas in athletes, we decided to choose the MNI coordinates of Buckner and colleagues [26] since the coordinates of all eight required ROIs come from the same study.

First, potential shape differences in the hand and foot representations between sportswomen and nonsportswomen will be taken into account and adjusted for by the nonlinear spatial transformations applied during preprocessing. Second, by way of comparison, in the study of Wenzel and colleagues [20], for example, the fMRI analysis of fast foot movements revealed activity in M1 with local maxima at *x* = 6, *y* = −36, and *z* = 70, which is comparable with the coordinates used for our ROI of FOOT_M1/R_ with *x* = 9, *y* = −26, and *z* = 76 and suggests that translocations (shifts) of these functional areas have not been expected. Third, by way of visual inspection we are able to show that the hand ROIs used to extract GM values do fully cover the omega-like shaped primary motor hand representation in our athletes.

These ROIs were used for the extraction of GM volume based on T1-weighted MRI scans but also served as seeds and targets in the probabilistic fibre tractography of the CST from these ROIs to the brainstem and from the brainstem to these ROIs (see below) based on DTI data.

### 2.5. Diffusion Tensor Imaging and Probabilistic Fibre Tractography

DTI data preprocessing and probabilistic fibre tractography were performed with FSL version 5.0.5. In a first step, nonbrain tissue was automatically removed using FSL's brain extraction tool. Further automated preprocessing steps (eddy-current and head movement correction) as well as the construction of individual diffusion tensor, FA, axial diffusivity (AD), and radial diffusivity (RD) maps were performed using FSL's diffusion toolbox 2.0 [38]. Even though our hypothesis is only referred to alterations in FA, we nonetheless analysed RD and AD in order to support the interpretation of potential significant FA findings. FA maps are scaled between 0 (fully isotropic diffusion) and 1 (fully anisotropic diffusion). DTI data was then prepared for probabilistic tractography using FSL's diffusion toolbox function called bedpostX (Bayesian estimation of diffusion parameters obtained using sampling techniques) with default parameters [39].

Using the above defined eight ROIs (FOOT_M1/R_, FOOT_M1/L_, FOOT_S1/R_, FOOT_S1/L_, HAND_M1/R_, HAND_M1/L_, HAND_S1/R_, and HAND_S1/L_) and an additional planar ROI placed on the height of the brainstem (MNI coordinate *z* = −22) tracking was performed from each cortical ROI (seed mask) to the brainstem ROI (waypoint and termination mask). An exclusion mask was set for the homotopic ROI in order to exclude fibres that might run across the corpus callosum to the opposite hemisphere. Default tracking parameters were used. Resulting tracts were in MNI space. After fibre tractography, within-tract probabilistic values were normalised at the individual level by dividing the number of streamlines passing through each voxel by the total number of obtained streamlines (“waytotal”) [40]. Subsequently, statistically normalised tracts were set at a threshold of 0.05 incorporating only those voxels where at least 5% of the total number of streamlines passed. Final tract maps were binarised and back-transformed from MNI into subjects' native space using inverse linear transformation and were used as subject-specific masks for extraction of FA, AD, and RD using the FSL functions “fslstats” and “fslmaths.”

Due to the fact that some voxels representing CST were included in both tracts, that is, in the reconstructed fibres running to the hand areas as well as in those running to the foot areas, these overlapping voxels were excluded in order to obtain FA, AD, and RD values that are associated with fibres connecting either the hand representations or fibres connecting the foot representations without any overlap of fibres connecting both representations.

### 2.6. Statistical Analyses

#### 2.6.1. Demographic Indices and Global Brain Measures

For the comparisons of demographic indices and global brain measures between ballet dancers, handball players, and controls, one-way analysis of variance (ANOVAs) models using IBM SPSS Statistics software version 22 (http://www-01.ibm.com/software/analytics/spss/products/statistics/) were applied. Error probability was set at *p* < 0.05.

#### 2.6.2. Analysis of Grey Matter Volume and Fractional Anisotropy

After extracting the mean GM volume of each of the eight ROIs (VBM analysis) and the mean FA, RD, and AD values, respectively, of each of the eight CST tracts (DTI analysis), these data was analysed using IBM SPSS Statistics software version 22. All data was tested for normality using the Kolmogorov-Smirnov test. We performed multifactorial, multivariate analyses of variance for repeated measures (rmMANOVAs) for the dependent variable GM volume as well as for FA, RD, and AD. We constructed both a 2 × 2 × 2 × 2 design with the between-subjects factor GROUP (ballet, handball) and, in order to additionally include a control group, a 3 × 2 × 2 × 2 design with the between-subjects factor GROUP (ballet, handball, and control) and the within-subjects factors BODYPART (hand, foot), MODALITY (M1, S1), and HEMISPHERE (right, left), respectively.

These analyses were performed once with the absolute ROI values and once with relative ROI values, that is, with normalised values. For the relative values, the absolute ROI values were divided by a global measure. For ROI GM volume, we used total global GM volume and for ROI FA, RD, and AD we used mean global FA, RD, and AD, respectively. In case of significant differences between the groups Tukey's HSD post hoc tests were applied.

Only to support the rmMANOVA findings, *t*-tests for independent samples were additionally performed between the handball players and the ballet dancers. Pearson correlations were used to associate the GM and FA findings with age of training commencement and years of training to associate the GM changes with the FA changes. Both *t*-tests and Pearson correlations were corrected for multiple comparisons by means of the false discovery rate (FDR). Effect sizes are reported based on the correlation coefficient *r* (correlations), Cohen's *d* (*t*-tests), and partial eta-squared (*η*_*p*_^2^; rmMANOVAs). According to Cohen, effect sizes are denoted small if *r* = 0.10, *d* = 0.20, and *η*_*p*_^2^ = 0.01, denoted moderate if *r* = 0.30, *d* = 0.50, and *η*_*p*_^2^ = 0.06, and denoted large if *r* = 0.50, *d* = 0.80, and *η*_*p*_^2^ = 0.14.

## 3. Results

### 3.1. Demographic Characteristics and Behavioural and Global Brain Measures

Demographic, behavioural, and global brain measures of the ballet dancers, handball players, and control women are summarised in Table 2. There were no significant differences between handball players, ballet dancers, and control women with respect to age, height, total GM, WM, cortical GM, and intracranial volume and also no difference in mean FA. Yet, post hoc tests revealed that handball players were on average significantly taller than ballet dancers (*p* = 0.049). With respect to mean RD and AD there were significant differences between the groups (RD: *F*_(2,41)_ = 4.53, *p* = 0.017; AD: *F*_(2,41)_ = 4.47, *p* = 0.018). However, as post hoc tests revealed, these significant differences are attributable to greater mean values in control women compared to handball players (RD: *p* = 0.012; AD: *p* = 0.013), whereas ballet dancers compared with both controls (RD: *p* = 0.703; AD: *p* = 0.414) and handball players (RD: *p* = 0.182; AD: *p* = 0.375) did not differ significantly. Furthermore, there was no significant difference between handball players and ballet dancers with respect to years of training, but compared to the handball players, the ballet dancers started training significantly earlier (*F*_(1,20)_ = 7.69, *p* = 0.012).

Moreover, compared with the handball players and the controls, the dancers showed significantly lower weight (*F*_(2,41)_ = 11.55, *p* < 0.001) and therefore their body mass index (BMI) is significantly lower than the one of the handball players and the controls (*F*_(2,41)_ = 9.02, *p* = 0.001). In contrast, handball players showed no significant difference with respect to the BMI compared to control women (*p* = 0.646) (Table 2).

### 3.2. Voxel-Based Morphometric Findings: Grey Matter Volume

All ROI GM volume data were approximately normally distributed except FOOT_M1/R_ of the ballet dancers (*p* = 0.020) and HAND_S1/R_ of the controls (*p* = 0.003). The results of the rmMANOVAs derived from the relative GM volumes (normalised by global GM volume) were not different from those derived from the absolute GM volumes and therefore only the results of the absolute GM volumes analyses are reported. Furthermore, given the lower BMI values of the ballet dancers compared to the other two groups, we also controlled for the BMI in an additional analysis, and since there was no single statistically significant effect of any factor in which the BMI is integrated, we did not include the BMI as a covariate in our statistical models (neither in GM nor in FA/RD/AD rmMANOVAs) in order to preserve statistical power. Other significant main effects or interactions than those relevant for our hypotheses will be reported in Results as they might be of some interest for future investigations, but they will not be discussed further.

**Figure 1:**
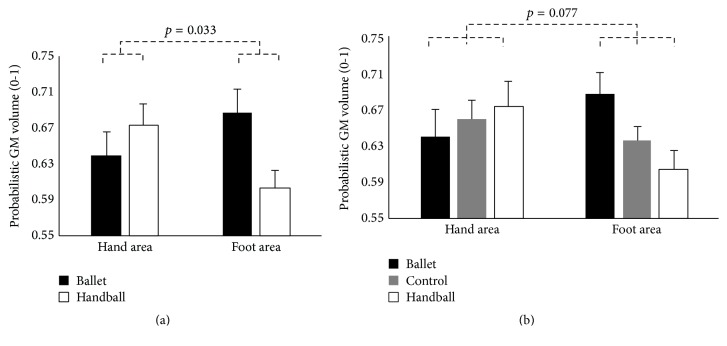
Estimated means and standard errors of GM volume of the hand and foot areas (independent of hemisphere and modality) for the ballet dancers (*n* = 10) and the handball players (*n* = 12) depicted on (a) (rmMANOVA including only the sports groups) and for the ballet dancers (*n* = 10) and the handball players (*n* = 12) and the control women (*n* = 22) depicted on (b) (rmMANOVA including all three groups). Note that increased GM reflects sport-specific skills.

Beside the main effect of MODALITY (2 groups: *F*_(1,20)_ = 16.51, *p* = 0.001, and *η*_*p*_^2^ = 0.452; 3 groups: *F*_(1,41)_ = 21.73, *p* < 0.001, and *η*_*p*_^2^ = 0.346) showing increased GM volume in S1 compared with M1, the 2-group rmMANOVA revealed the predicted interaction of GROUP x  BODYPART (*F*_(1,20)_ = 5.27, *p* = 0.033, and *η*_*p*_^2^ = 0.208). This interaction indicated increased GM volume in the feet representations of ballet dancers compared to handball players, whereas handball players showed increased GM volume in the representations of the hands compared with ballet dancers. With respect to the 3-group rmMANOVA including the control group the predicted interaction of GROUP x BODYPART was shown only with a trend towards significance (*F*_(2,41)_ = 2.74, *p* = 0.077, and *η*_*p*_^2^ = 0.118) (Figure 1, Table 3).

Another significant group-specific interaction was also found between the factors GROUP x HEMISPHERE (*F*_(1,20)_ = 7.11, *p* = 0.015, and *η*_*p*_^2^ = 0.262) and with a trend towards significance as including the control group (*F*_(2,41)_ = 2.85, *p* = 0.069, and *η*_*p*_^2^ = 0.122), showing increased GM volume in the left hemisphere in handball players compared with ballet dancers (and controls, resp.), whereas right-hemispheric GM volume was increased in ballet dancers compared with handball players (and controls, resp.).

A further significant but not group-specific interaction was found for the factors HEMISPHERE x BODYPART (*F*_(1,20)_ = 8.48, *p* = 0.009, and *η*_*p*_^2^ = 0.298; *F*_(1,41)_ = 9.05, *p* = 0.004, and *η*_*p*_^2^ = 0.181) showing that GM volume was increased in the right hand areas compared with the left hand areas, whereas GM volume in the left foot areas was increased compared with the right foot areas.

The findings of the additionally conducted *t*-tests for independent samples are summarised in Table 3, but these findings only partially support the predicted interaction found between the factors GROUP x BODYPART. The average GM volume of FOOT_M1/R_ (*t*_(20)_ = 3.66, *p* = 0.016, and *d* = 1.64) and FOOT_S1/R_ (*t*_(20)_ = 3.17, *p* = 0.020, and *d* = 1.42) is increased in ballet dancers compared with handball players. This supports the predicted interaction of GROUP x BODYPART in the sense that GM volume is increased in the feet representations of ballet dancers compared with handball players, but statistical significance was only reached for the right but not the left foot area, which in turn is in line with the interaction found between GROUP x HEMISPHERE, which showed that ballet dancers compared with handball players have increased GM volume in the right-hemispheric ROIs. As can be seen in Table 3, the mean values, with the exception of FOOT_M1/L_ and HAND_S1/R_, nevertheless support the interaction because the feet representations showed with large effect sizes increased GM volume in ballet dancers compared with handball players, whereas GM volume was increased in the hand areas in handball players compared with ballet dancers.

The mean GM volumes of each ROI were then correlated with age of training commencement and with the number of years of training within each sports group separately. In handball players, the GM volume of HAND_S1/R_ correlated positively with the number of years of training (*r* = 0.790, *p* = 0.002) and that of FOOT_M1/R_ correlated inversely with the age at training commencement (*r* = −0.720, *p* = 0.008). No further statistically significant correlations were found.

### 3.3. Diffusion Tensor Imaging Findings: Fractional Anisotropy

**Figure 2:**
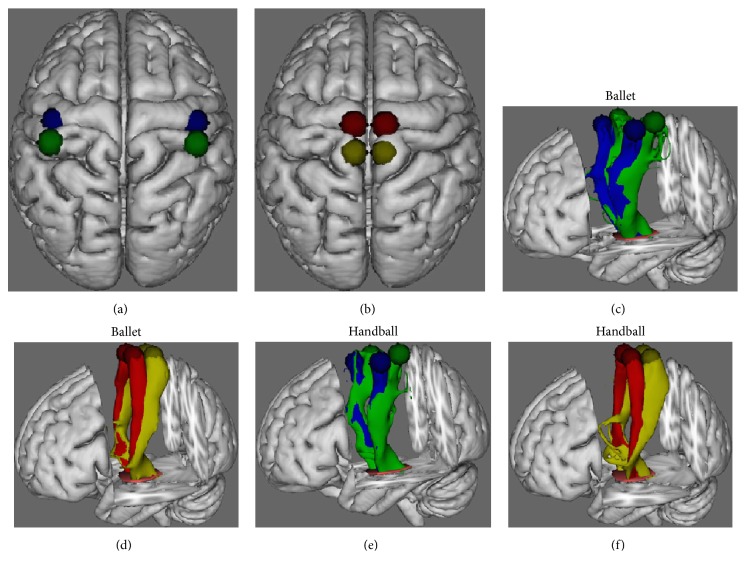
Probabilistic reconstruction of the corticospinal tracts (CSTs). (a) shows both, the spherical masks used for the VBM and the DTI analysis of the predefined hand areas in M1 (blue) and S1 (green) and (b) of the predefined foot areas in M1 (red) and S1 (yellow). (c–f) Probabilistic reconstruction of the WM fibres representing the mean CSTs of all ballet dancers (c and d) and all handball players (e and f), respectively, between the left- and the right-hemispheric hand areas (blue: M1, green: S1) and the left- and the right-hemispheric foot areas (red: M1, yellow: S1) and the brainstem (orange).

The ROIs used for GM volume extraction and fibre tractography are presented in Figures 2(a) and 2(b) and the reconstructed CSTs are shown in Figure 2(c) (hands) and Figure 2(d) (feet) for the ballet dancers and in Figure 2(e) (hands) and Figure 2(f) (feet) for the handball players. Blue and red represent the connections of the primary motor areas whereas green and yellow represent the connections of the primary somatosensory areas.

All ROI FA data was approximately normally distributed. The results of the rmMANOVA derived from the relative FA values (normalised by mean global FA) were not different from those derived from the absolute FA values and therefore only the absolute FA values are reported. Again, other significant main effects or interactions than those relevant for our hypotheses will be reported as they might be of some interest for future investigations but they will not be discussed further.

Beside a significant main effect of the factor MODALITY (*F*_(1,20)_ = 81.70, *p* < 0.001, and *η*_*p*_^2^ = 0.803 and *F*_(1,41)_ = 112.11, *p* < 0.001, and *η*_*p*_^2^ = 0.732, resp.) revealing decreased FA in S1 compared with M1, we found a significant main effect of the factor GROUP (*F*_(1,20)_ = 26.91, *p* < 0.001, and *η*_*p*_^2^ = 0.574 and *F*_(2,41)_ = 9.81, *p* < 0.001, and *η*_*p*_^2^ = 0.324, resp.) with decreased FA in ballet dancers compared to handball players. The follow-up post hoc comparisons with respect to the 3-group analysis revealed decreased FA in ballet dancers both compared to handball players (*p* < 0.001) and controls (*p* = 0.018), whereas handball players showed only marginally increased FA compared to controls (*p* = 0.077).

**Figure 3:**
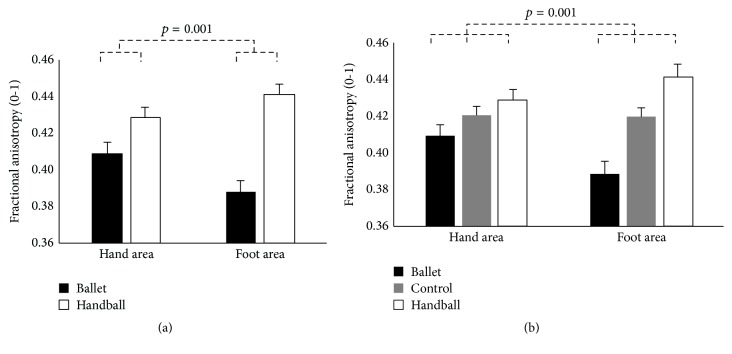
Estimated means and standard errors of fractional anisotropy (FA) in the corticospinal tracts originating in the hand and foot areas (independent of hemisphere and modality) for the ballet dancers (*n* = 10) and the handball players (*n* = 12) depicted on (a) (rmMANOVA including only the sports groups) and for the ballet dancers (*n* = 10) and the handball players (*n* = 12) and the control women (*n* = 22) depicted on (b) (rmMANOVA including all three groups). Note that reduced FA reflects sport-specific skills.

Furthermore, we detected the predicted interaction between the factors GROUP x BODYPART (*F*_(1,20)_ = 16.66, *p* = 0.001, and *η*_*p*_^2^ = 0.454 and *F*_(2,41)_ = 7.74, *p* = 0.001, and *η*_*p*_^2^ = 0.274, resp.) (Figure 3, Table 4). These interactions, however, indeed revealed decreased FA in both the fibres connecting the hand and foot areas in ballet dancers compared to handball players (and controls, resp.), but ballet dancers showed lower FA in the fibres connecting the foot compared to their hand areas, whereas handball players showed lower FA in the fibres connecting the hand compared to their foot areas.

**Figure 4:**
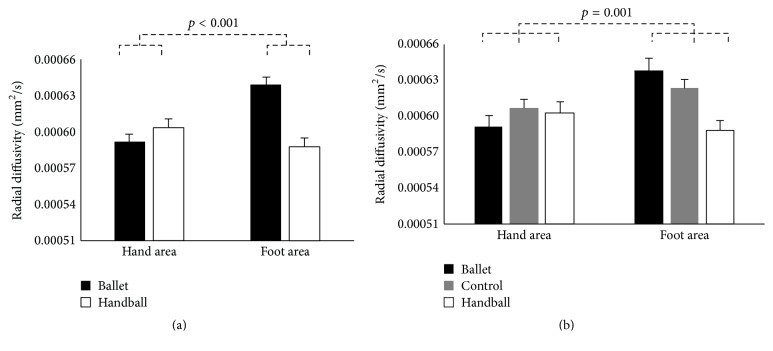
Estimated means and standard errors of radial diffusivity (RD) in the corticospinal tracts originating in the hand and foot areas (independent of hemisphere and modality) for the ballet dancers (*n* = 10) and the handball players (*n* = 12) depicted on (a) (rmMANOVA including only the sports groups) and for the ballet dancers (*n* = 10), the handball players (*n* = 12) and the control women (*n* = 22) depicted on (b) (rmMANOVA including all three groups). Note that increased RD reflects sport-specific skills.

As mentioned above, we also analysed RD and AD to support the interpretation of a potential significant interaction of FA of GROUP x BODYPART. With respect to AD this interaction did not reach statistical significance neither for the 2 groups (*F*_(1,20)_ = 3.32, *p* = 0.084, and *η*_*p*_^2^ = 0.142) nor for the 3 groups (*F*_(2,41)_ = 2.34, *p* = 0.109, and *η*_*p*_^2^ = 0.102) rmMANOVA. The RD analysis, however, revealed a significant interaction of GROUP x BODYPART (*F*_(1,20)_ = 24.45, *p* < 0.001, and *η*_*p*_^2^ = 0.550 and *F*_(2,41)_ = 8.09, *p* = 0.001, and *η*_*p*_^2^ = 0.283, resp.), showing, contrary to FA, increased RD in the fibres connecting the foot areas in ballet dancers compared to handball players (and controls) but, as in FA, decreased RD in the fibres connecting the hand areas in ballet dancers compared to handball players (and controls). However, considering the differences within the groups, the ballet dancers showed higher RD in the fibres connecting the foot compared to their hand areas, whereas handball players showed higher RD in the fibres connecting the hand compared to their foot areas, where both correspond to the opposite directions of FA (Figure 4, Table 5).

A further significant group-specific interaction was found in GROUP x HEMISPHERE (*F*_(1,20)_ = 87.96, *p* < 0.001, and *η*_*p*_^2^ = 0.815 and *F*_(2,41)_ = 21.94, *p* < 0.001, and *η*_*p*_^2^ = 0.517, resp.) revealing decreased FA both in the part of the CST connecting left- and right-hemispheric ROIs in ballet dancers compared to handball players (and controls); whereas for handball players FA is decreased in the part of the CST connecting the right- compared to their left-hemispheric ROIs, FA in ballet dancers (and controls) is lower in the part of the CST connecting the left- compared to their right-hemispheric ROIs.

The findings of the additionally conducted *t*-tests for independent samples are summarised in Table 4 for FA and in Table 5 for RD, respectively. In four of the eight investigated tracts significant group differences in FA were found, which is in line with the interaction between the factors GROUP x HEMISPHERE as revealed by the rmMANOVA. All parts of the CST investigated in the left hemisphere (HAND_M1/L_, HAND_S1/L_, FOOT_M1/L_, and FOOT_S1/L_) showed statistically significantly decreased FA values with large effect sizes in ballet dancers compared with handball players (Table 4). In RD also four of the eight investigated tracts showed significant group differences. Three of it concern the foot areas (FOOT_M1/L_, FOOT_S1/L_, and FOOT_M1/R_) showing significantly increased RD in ballet dancers compared to handball players and one concerns the hand area (HAND_M1/R_) with significantly increased RD in handball players compared to ballet dancers (Table 5).

Finally, the mean FA and RD values of each ROI were then correlated with age of training commencement and with the number of years of training within each sports group separately. No statistically significant correlations were found.

### 3.4. Associations between Grey Matter Volume and Fractional Anisotropy

For reasons of clarity and comprehension the Pearson correlations used to associate the GM and FA findings were conducted with only four instead of the initial eight ROIs. The ROIs were averaged with respect to the hemispheres because a significant main effect for the factor MODALITY was found, whereas, for the factor HEMISPHERE, this was not the case (HAND_M1/R+L_, HAND_S1/R+L_, FOOT_M1/R+L_, and FOOT_S1/R+L_).

In ballet dancers, the following associations were significant or showed a trend towards significance, respectively: between GM-HAND_S1/R+L_ and FA-FOOT_M1/R+L_ (*r* = 0.69, *p* = 0.042) and between GM-FOOT_M1/R+L_ and FA-HAND_S1/R+L_ (*r* = 0.57, *p* = 0.087).

In handball players, the following associations were significant or showed a trend towards significance, respectively: between GM-HAND_M1/R+L_ and FA-HAND_S1/R+L_ (*r* = −0.69, *p* = 0.028), GM-HAND_M1/R+L_ and FA-FOOT_M1/R+L_ (*r* = −0.70, *p* = 0.036), and GM-HAND_M1/R+L_ and FA-FOOT_S1/R+L_ (*r* = −0.85, *p* = 0.003) as well as between GM-HAND_S1/R+L_ and FA-HAND_S1/R+L_ (*r* = −0.57, *p* = 0.070).

## 4. Discussion

As predicted, we found structural brain differences in the a priori defined brain regions between professional handball players and ballet dancers. The main result can be considered in the fact that GM volume in the hand representations is increased in handball players compared with ballet dancers, whereas GM volume in the foot representations is increased in ballet dancers compared with handball players. However, as including the control group, this interaction was only shown with a trend towards significance. The larger GM volume in the hand area of the handball players is supported by their positive correlation between GM volume in HAND_S1/R_ and the number of years of training suggesting that the GM volume in the hand area is enhanced with increasing years of training. This double dissociation is additionally supported by the differences in mean GM volume, for which handball players showed increased volume in three out of the four hand areas investigated (M1/S1 and left/right), whereas ballet dancers showed increased GM volume in three out of the four foot areas investigated. Indeed, not all of these differences in mean GM volume reached statistical significance, but all showed medium to large effect sizes, which leads to the assumption that a larger sample size would cause these results to reach statistical significance.

With respect to FA, the hypothesis was not answered as clearly. There is indeed a significant interaction between the factors GROUP x BODYPART, but ballet dancers showed decreased FA in both the fibres connecting the foot and hand areas compared to handball players. The fact that ballet dancers showed lower FA in the fibres connecting the foot areas compared to their hand areas, whereas handball players showed lower FA in the fibres connecting the hand areas compared to their foot areas, nevertheless strongly supports our hypothesised interaction of GROUP x BODYPART. Moreover, given that the significant interaction of GROUP x BODYPART in RD revealed exactly the opposite alterations compared with the interaction found in FA (in ballet dancers higher RD in the fibres connecting the foot compared to their hand areas and in handball players higher RD in the fibres connecting the hand compared to their foot areas), it provides evidence that the reduced FA is driven by increased RD suggesting changes in myelinisation rather than changes in the axonal membrane [41].

### 4.1. Differences in the Hand and Foot Representation

The findings in the GM volume, which was increased in the hand areas in handball players and increased in the foot areas in ballet dancers (interaction GROUP x BODYPART), suggest that training-induced neuroplastic adaptations are actually sport-specific rather than just sport-general. The results found as predicted are astonishing considering the fact that although the demands on the feet/legs are stronger in ballet dancers and the demands on the hands/arms are stronger in handball players, both sports need the upper and lower extremities intensively. Our results strongly suggest that brain structures differ in accordance with the demands and skills needed for a particular sport, although the two groups of athletes do not differ at all in their sportive experience and training intensity.

As already mentioned, there are only three studies we are aware of comparing two groups of athletes [16, 17, 20]. However, even though all of these studies revealed structural changes in specific brain regions between the athletes when compared with nonathletes, only the studies conducted by Schlaffke [17] and Wenzel [20] and colleagues, respectively, revealed also brain structures adapted in one sport group but not in the other. These results as well as the findings of the present study suggest that the neuroplastic adaptations observed (here an increase in GM volume) are sport-specific and therefore take place in different brain areas.

Increased GM volume in the hand area of handball players compared with ballet dancers is supported by another study, of course using the same sample of handball players, which revealed increased GM volume in the bilateral M1 and S1 hand representation when professional female handball players were compared with control women [13]. A further study investigated experience-dependent plasticity of the motor hand representation in blind subjects who were able to read Braille and revealed that the representation of the index finger of the reading hand is increased compared to that of the nonreading hand and also in comparison with control subjects who were not blind [42]. Granert and colleagues [43] investigated right-handed subjects who suffer from the writer's cramp (chirospasm, also known as focal hand dystonia) and showed that four weeks of immobilisation of the affected hand leads to GM volume decreases in the contralateral M1 hand area, whereas subsequent eight weeks of training of the affected hand leads to GM volume increases in the contralateral M1 hand area [43]. Retrospective studies on professional musicians provide further evidence for our findings. Elbert and colleagues [44] reported that the representations of the fingers of the left hand are significantly increased in musicians playing string instruments compared with nonmusicians [44]. In a human deprivation study, our group investigated ten right-handed subjects who suffer from injuries of the right upper extremity that need an immobilisation of the right arm for about 14 days. MRI scans were acquired within 48 h after injury and after an average immobilisation period of 16 days and showed a decrease in cortical thickness in the left M1 and S1 hand area and a significant reduction in FA in the left CST. In addition to these deprivation-induced reductions in cortical thickness and FA in contralateral brain regions, the performance of motor skills of the left nondominant noninjured hand improved during the immobilisation period and cortical thickness of the right M1/S1 hand representation increased as well [45]. Makin and colleagues [46] investigated cortical plasticity following congenital or acquired hand absence and found that adaptive limb-usage strategies drive both functional and structural plasticity in adult humans. The authors found that the deprived cortex was employed by whichever part of the upper limb individuals were using to compensate for their impairment, independently of which specific body part was affected and to what degree this part was typically represented in the respective cortical area [46].

The increased GM volume in the cortical representation of the foot area of ballet dancers compared with handball players and control women shows that training-induced structural adaptations are not restricted to the representation of the hand. To the best of our knowledge, we are not aware of any study that investigated the long-term impact of somatosensory-motor training of feet and legs. However, there are findings that can be related to our results. Liepert and colleagues [47] showed that the immobilisation of a foot during four to six weeks due to a bone fracture leads to a decrease in the cortical representation of that foot. The time of immobilisation was directly related to the amount of the decrease in the cortical representation [47]. It has also been shown that the cortical motor excitability of the foot/leg area is increased after a 32-minute foot movement training compared with a passive as well as compared with a non-foot-specific training [48]. In a functional MRI study, Naito and Hirose [49] investigated the activity in the foot area during foot movements in soccer players including the famous Brazilian soccer player Neymar Jr. In contrast to other professional and amateur soccer players and also in contrast to professional swimmers, Neymar Jr. showed reduced activity in his foot area during simple foot movements, a reduction in activity which has been interpreted as a sign of neural efficiency [49]. This result is in line with findings from the neuroplastic literature about the hand area in musicians. Professional musicians compared with nonmusicians repeatedly showed reduced activity in the hand area when performing simple finger movements, which was also interpreted as a sign of neural efficiency [50, 51].

The interaction of GROUP x BODYPART of FA values was statistically significant but did not directly show the hypothesis-conform pattern of differences (decreased or increased FA in the hand areas of handball players compared to decreased or increased FA in the foot areas of ballet dancers). Instead, ballet dancers showed decreased FA in both the part of the CST connecting the foot area and the one connecting the hand area compared with handball players and control women. Since in previous studies training-induced neuroplastic adaptations were observed in both directions (decreases and increases) and not only in WM but also in GM (for an overview see Table 1 of [13]), it remains unclear whether our results have to be interpreted as decreased FA in ballet dancers compared to handball players or as increased FA in handball players compared to ballet dancers. However, when assuming reduced FA instead of increased FA as a sign of sport-specific neuroplastic adaptations, the fact that ballet dancers showed lower FA in the fibres connecting the foot compared to their hand areas, whereas handball players showed lower FA in the fibres connecting the hand areas compared to their foot areas, would be in line with our hypothesis and supports the idea that the foot areas are selectively modulated by ballet dancing, whereas the hand areas are selectively modulated by playing handball. This assumption is further supported by the findings with respect to RD showing exactly the opposite direction of neuroplastic changes, for example, in ballet dancers increased RD in the fibres connecting the foot compared to their hand areas and in handball players increased RD in the fibres connecting the hand compared to their foot areas, respectively. These results provide evidence that reduced FA is driven by increased RD suggesting changes in myelinisation rather than changes in the axonal membrane [41].

In line with the assumption of reduced FA as a sign of training-related neuroplastic adaptations are the findings of the former ballet study of our group, in which the same ballet dancers showed reduced FA values in the premotor cortex near the primary motor cortex when compared with nonathletes [19]. Furthermore, right-handedness per se is associated with reduced FA in the left compared with the right CST, not only in professional musicians but also in nonmusicians. When musicians were contrasted against nonmusicians, the former showed reduced FA in both CSTs compared with the latter [52], although musicians commonly outperform nonmusicians in classical finger tapping task [50, 51]. With respect to the CST, it was highlighted that professional golf players showed reduced FA in their CSTs [18], whereas professional world-class gymnasts showed increased FA in their CSTs [53]. However, the majority of studies published so far suggest that high performance in a particular sport is rather associated with an increase in GM and WM volume as well as FA.

In the field of functional neuroplastic adaptations however, it is accepted that specific skills in a domain are associated with reduced neuronal activity in brain regions involved in the control of the specific expertise [50, 51, 54–56]. As already mentioned, such activity reductions were also reported for the professional soccer player Neymar Jr. [49], one of the best soccer players nowadays. In our opinion, these expertise-related activity reductions might be accompanied by local reductions in GM and WM volume as well as in FA as a consequence of a long-term intensive training and expertise [19].

A further explanation for the contradictory findings with respect to the direction of the adaptations (decreases or increases) might be related to the training phase in the sense that anatomical alterations in the form of increases take place in a rather early phase of training, whereas in later training stages no further increases or even decreases occur [19]. In one of the aforementioned juggling studies [9], it was shown that GM volume increases were observable mainly in the early phase of juggling training (here after seven days) but not in later phases. Indirect support for this explanation can also be derived from a study that investigated structural brain correlates of golf playing and revealed no differences in brain structures between professional and amateur golf players, although these two groups differ considerably in the retrospectively estimated total amount of lifetime spent for golf training (mean/SD: 27,415/12,542 hours in professionals and 3,207/2,916 hours in amateurs) [18]. It is an ongoing discussion, if observed cortical plasticity, that is, in GM volume in ballet dancers or handball players, can be interpreted as a result of long-term intense training or if it represents one reason why an individual even dedicates him/herself to a specific field. In an interesting recently published study, Sampaio-Baptista and colleagues [57] established a link between baseline GM volume and subsequent juggling performance after six weeks of practice. Indeed, they not only were able to show that performance outcome and the amount of practice modulated structural plasticity, but also highlighted that interindividual baseline differences in GM volume correlated with performance outcome; that is, greater GM volume in certain areas of the brain was related to steeper learning slopes [57].

A third possibility might be rooted in interactional effects between the elements of a skill and the resulting anatomical adaptations. It is conceivable that the learning and training of a particular skill are associated with anatomical adaptations, irrespective of whether GM volume and FA increases or decreases occur, whereas the training of other skills might evoke no anatomical adaptations at all [19]. The understanding of such interactions might be further complicated by other training-related variables such as training duration, stage of training, training strategies, and age of onset [19]. Referring to the latter, Steele and colleagues [58] showed that early musical training (training onset before the age of 7 years) had a differential impact on WM structure providing evidence for a sensitive period where experience produces long-lasting changes in the brain and behaviour. Moreover, also Vaquero and colleagues [59] found an association between age of onset and structural brain changes; for example, early onset of piano playing was associated with smaller GM volume in the right putamen as well as with better piano performance. However, considering the current GM findings, it was shown that both groups revealed the predicted brain alterations and also that these alterations were in the same direction, although the ballet dancers started training much earlier than the handball players (ballet dancers with an average of 7.3 years compared to 10.58 years in handball players). Furthermore, we correlated the age of training onset with the GM, FA, and RD values of the eight ROIs across both groups as well as within each group separately. With respect to GM, no significant correlations were found within the ballet dancers and only one significant correlation (FOOT_M1/R_) was revealed within the handball players (*r* = −0.720, *p* = 0.008), a correlation also found across both groups (*r* = −0.721, *p* = 0.0002). Moreover, age of training onset correlated positively with FA in FOOT_M1/L_ (*r* = 0.510, *p* = 0.01) and FOOT_S1/L_ (*r* = 0.433, *p* = 0.044), respectively, whereas RD within these ROIs was inversely related to age of training onset (*r* = −0.572, *p* = 0.005 and *r* = −0.521, *p* = 0.011, resp.). Therefore, these correlations only provide weak evidence for the idea that age of training onset is an important factor in order to explain the neuroplastic alterations observed in the ballet dancers and handball players of the current study.

Finally, the neuroplastic adaptations might also be prone to specific biological circumstances such as the genetic background or the eating behaviour that might interact with brain developmental processes [19]. Particularly ballet dancers showed on average a significantly lower BMI compared with the handball players.

Potential reasons for the inconsistent FA findings can be numerous and are still not investigated at all. Due to the fact that FA is the ratio between AD and RD, high FA values reflect stronger AD along the fibres. Reduced FA can reflect increased RD, decreased AD, or a mixture of both. However, reduced FA can also be observed if different fibres cross, bend, and/or twist within a voxel. This is because the classical DTI tensor model can measure only one fibre direction per voxel. Diffusion spectrum imaging (DSI) might help disentangling whether there are indeed reduced FA values in a voxel or whether different fibres cross, bend, and/or twist within that voxel [19]. However, this is only a speculation and further studies are needed in order to investigate training-induced adaptations in the white matter fibre bundles.

### 4.2. Associations between Grey Matter Volume and Fractional Anisotropy

The aim of the analysis of associations between the two morphometric measures investigated was to test whether adaptations in GM and FA are related to each other or whether they are independent of each other [13]. These associations were computed with the four averaged ROIs for each athlete group separately. The significant correlations found were all positive for ballet dancers, and these correlations were found between the GM volume of the hand areas and FA of CST fibres connecting the foot areas or vice versa. In contrast, all correlations found for the handball players were negative and were found between the GM volume of the hand areas and FA of CST fibres connecting the hand or the foot areas. These significant associations suggest that adaptations in one brain region are related to adaptations in other brain regions and it might therefore be possible that changes in one brain region influence changes in other brain regions and that the direction of such influences might be sport-specific. However, these speculations need further investigation using longitudinal study designs and complementary methods.

### 4.3. Neuroplastic Alterations Underlying Cellular Mechanisms

In order to make inferences about possible physiological consequences of cellular alterations, it is necessary to know what kind of microstructural cellular mechanisms underlies the macroscopic changes. However, by using MRI, structural neuroplastic alterations can only be measured at the macroscopic scale and therefore the underlying microscopic cellular events of these changes are not clarified yet. Nevertheless, conceivable microstructural mechanisms have been proposed, that is, an increase in cell size, genesis of new synapses, genesis of glial or even neural cells, or changes in spine density, blood flow, interstitial fluid, or even angiogenesis [60, 61]. Yet, the current knowledge with respect to these cellular and physiological mechanisms is still insufficient; hence it remains a future challenge to provide convincing explanations for the cellular changes underlying these macrostructural adaptations.

### 4.4. Implications

The fact that our results suggest sport-/skill-specific rather than just expertise-general neural adaptations as well as the fact that not only the hand but also the foot representations can be increased could probably play a role in contributing to the development of training-supportive methods for professional athletes. The aforementioned results of the studies conducted by Granert [43], Langer [45], and Liepert [47] and colleagues, respectively, additionally emphasize that a disuse of the upper and lower limbs can lead to a decrease in the corresponding cortical representation.

The mental imagination of motoric movements, called motor imagery (MI) [62], could be a potential method. Brain activity measurements have proven that the imagination of motoric actions can trigger activity in similar cortical networks such as the real motion execution [63]. Stippich and colleagues [64] revealed that MI of different body parts (foot, hand, and tongue) activates the precentral gyrus in a somatotopic way. This indicates, as our results do, that both the cortical hand and foot representation can be influenced by means of training. Given that functional adaptations in consequence of MI were shown in healthy subjects [65], it is conceivable that this method could be adapted to athletes as well. So far, the attention of mental training science has been focused on mental training in the sense of mental preparation for upcoming achievements/competitions rather than on MI [66]. Weinberg [66], however, provided evidence for performance-enhancing effects in consequence of MI. Moreover, in a study of Wei and Luo [67] it was shown that MI of specific movements also differs on a neuronal level between experts and nonexperts. The authors revealed that professional divers exhibit higher activity in the parahippocampus during MI of specific high diving movements compared to both control subjects and during MI of simple movements like walking, jumping, or hand movements. Further, with respect to the latter there was no difference in neural activity in divers compared to controls [67]. Moreover, numerous studies (for an overview see [68]) showed that, due to MI, brain activity also changed in areas of the motor cortex, such as premotor cortex, supplementary motor area, or primary motor cortex. Particularly with regard to the investigated ballet dancers and handball players in the current study it would be interesting to examine whether MI has an effect on adaptations in cortical representations and on functional skills, respectively, given the fact that the limbs, which are most important for their success, also have been reflected in changes of the cortical representation. If true, MI would be an easy and everywhere practicable method which can be fitted well into the physical training. For instance, potentially technical deficits of certain dancing steps or of catching and throwing a ball, respectively, could possibly be trained additionally by means of MI and hence improve the real execution of the movement, which needs perfecting. Beyond that, MI could also be used in case of hand/foot injuries to compensate for the training absence and hence for a functional deficit as well as to prevent or at least minimize a potential decrease of the cortical representations, which in turn would entail some advantages with regard to the comeback into training after injury layoff.

### 4.5. Limitations

Several limitations of the present study are worth mentioning. First, the findings of the present study should not be considered to represent a fully independent replication of the findings of our two already published studies [13, 19] due to the fact that neither the experimental subjects investigated here were independent of those investigated in the two previous studies nor were the contrasts of the present study orthogonal to those of the previous studies. Second, although the sample sizes were rather small (10 professional ballet dancers, 12 professional handball players, and 22 controls) and hence statistical power is limited, the effects reported showed medium to large effect sizes leading to the assumption that increasing sample sizes would simply cause actual results to shift towards even greater effect sizes. Third, whether the structural alterations found in handball players and ballet dancers are the result of training-induced neuroplastic adaptations (nurture) or stem from a genetic predisposition (nature) for a “ball playing affinity” and “dancing affinity,” respectively, should be investigated in future longitudinal studies. To date, we can only state that there are distinct structural differences with regard to upper and lower extremities (hands and feet) between handball players and ballet dancers despite the fact that both groups showed similar above-average levels of training and experience. Fourth, it remains unclear to which extent the results found in exclusively female samples can be generalized on male handball players and male ballet dancers, respectively. However, for keyboard players it has been reported that structural brain correlates of musicianship were more pronounced in male compared with female musicians and that the effect of musicianship in female keyboard players might be masked by the fact that females showed larger relative (% of total brain volume) cerebellar volumes in general [69]. Future studies should therefore investigate both sportsmen and sportswomen.

Last, a potentially critical aspect to address is the observed statistically significant difference between the two sports groups with regard to height, weight, and BMI. Last, ballet dancers showed considerably lower height, weight, and BMI as compared to handball players and control women, whereby 6 of the 10 ballet dancers would be considered slightly underweighted with BMIs below 18.5 kg/m^2^ (http://www.euro.who.int/en/health-topics/disease-prevention/nutrition/a-healthy-lifestyle/body-mass-index-bmi). Even though the calculation of the BMI does not distinguish between fat and muscle tissues and its relevance and interpretability when applied to athletes remain subject of dispute, a low BMI may indicate some form of malnutrition. Indeed, there is some evidence in connection with restrictive anorexia nervosa that malnutrition may lead to a reduction of GM volume in the brain. However, the findings were restricted to certain brain areas such as the anterior cingulate cortex, the frontal operculum, and temporoparietal regions [70]. None of these regions were included in our investigations since the focus lay on the hand and foot representation in the primary motor and somatosensory cortex. We therefore believe it to be unlikely that slight underweight of a few ballet dancers biased the results found in a significant way.

### 4.6. Conclusions

As predicted, sport-specific rather than sport-general adaptations in the M1 and S1 representation of the hand and feet were found between professional handball players and ballet dancers. The main GM volume finding is an interaction between the factors GROUP x BODYPART that revealed increased GM volume in the feet representations of ballet dancers compared with handball players, whereas handball players showed increased GM volume in the representations of the hands compared with ballet dancers. Furthermore, a statistically significant GROUP x BODYPART interaction was found for FA, but compared to handball players, ballet dancers showed decreased FA in both the fibres connecting the foot and hand areas. Nevertheless, there is an interaction in line with our hypothesis that ballet dancers showed lower FA in the fibres connecting the foot compared to their hand areas, whereas handball players showed lower FA in the fibres connecting the hand compared to their foot areas.

Our results suggest that brain structures differ in accordance with the demands and skills needed for a sport and therefore indeed speak for sport-specific rather than just sport-general neuroplastic adaptations. However, longitudinal studies are needed in order to unequivocally track down whether the observed structural alterations between handball players and ballet dancers are driven by nurture (training-induced neuroplasticity) or by nature (genetic predisposition) or potentially driven by interactions between both.

## Figures and Tables

**Table 1:** Coordinates of the regions of interest used for the primary motor and primary somatosensory representation of the hands and feet.

	MNI coordinates					
ROI	Right hemisphere			Left hemisphere		
	*x*	*y*	*z*	*x*	*y*	*z*
HAND_M1_	41	−20	62	−41	−20	62
HAND_S1_	42	−35	65	−42	−35	65
FOOT_M1_	9	−26	76	−9	−26	76
FOOT_S1_	9	−42	74	−9	−42	74

*Note*. Coordinates represent locations of peak voxels (cf. [26]) as centers for the construction of spherical masks with radius 8 mm. Due to overlapping spheres in the primary motor and primary somatosensory feet areas, the coordinates of the *x*-axis were slightly modified from originally 6 to 9 (right) and −6 to −9 (left) in M1 and 10 to 9 (right) and −10 to −9 (left) in S1, respectively. M1, primary motor cortex; S1, primary somatosensory cortex; MNI, Montreal Neurological Institute space; ROI, region of interest.

**Table 2:** Demographic characteristics and global brain measures of the female professional ballet dancers, professional handball players, and control women.

Measure	Professional ballet dancers (*n* = 10)				Professional handball players (*n* = 12)				Control women (*n* = 22)			
	*M*	SD	Min.	Max.	*M*	SD	Min.	Max.	*M*	SD	Min.	Max.
Age (years)	21.94	3.03	18.30	27.80	23.25	2.96	19.00	29.00	25.19	4.20	18.50	37.80
Body height (m)	1.67	0.06	1.57	1.75	1.72	0.04	1.67	1.81	1.70	0.05	1.60	1.82
Body weight (kg)	52.00	3.68	48.00	60.00	65.58	10.14	50.00	87.00	62.23	5.72	50.00	71.00
Body mass index (BMI)	18.68	1.53	16.96	21.91	22.15	2.56	17.93	27.46	21.49	1.91	18.59	25.77
Quantity of training (years)	14.20	3.26	10.00	20.00	12.67	2.67	7.00	16.00				
Age at training commencement	7.30	2.50	4.00	13.00	10.58	2.97	5.00	15.00				
Total GM volume (cm^3^)	619.34	64.75	523.40	723.52	644.00	40.39	580.05	704.55	633.82	39.92	552.94	714.04
Total WM volume (cm^3^)	454.13	48.58	378.11	522.32	474.06	41.40	410.44	568.33	479.92	29.87	420.33	517.56
Total cortical GM volume (cm^3^)	569.20	49.58	492.68	636.32	578.76	54.48	481.25	650.80	593.48	36.25	522.35	672.21
Total IC volume (cm^3^)	1288.48	120.67	1141.30	1459.84	1384.21	150.15	1044.42	1599.98	1371.90	128.01	1151.27	1648.63
Mean FA	0.24	0.01	0.22	0.24	0.24	0.01	0.22	0.26	0.24	0.01	0.23	0.26
Mean AD	0.00114	0.00003	0.00111	0.00120	0.00112	0.00004	0.00105	0.00118	0.00116	0.00003	0.00112	0.00122
Mean RD	0.00081	0.00003	0.00078	0.00086	0.00079	0.00004	0.00073	0.00084	0.00082	0.00003	0.00076	0.00089

*Note*. AD, axial diffusivity; FA, fractional anisotropy; GM, grey matter; IC, intracranial; *M*, mean; Max, maximum; Min., minimum; *n*, number of subjects; RD, radial diffusivity; SD, standard deviation; WM, white matter.

**Table 3:** Group comparisons of cortical volume (GM) in each region of interest (ROI).

ROI	Ballet (*n* = 10)		Handball (*n* = 12)		Controls (*n* = 22)		*t *(20)	*p*	*d*
	*M*	SD	*M*	SD	*M*	SD			
HAND_M1/R_	0.6140	0.0972	0.6567	0.0979	0.6412	0.1118	−1.02	0.510	−0.46
HAND_M1/L_	0.5510	0.0885	0.6129	0.0834	0.6031	0.1346	−1.69	0.285	−0.76
HAND_S1/R_	0.7329	0.1291	0.6862	0.0905	0.7083	0.1897	0.99	0.379	0.45
HAND_S1/L_	0.6605	0.1606	0.7341	0.1809	0.6825	0.1582	−1.00	0.440	−0.45
FOOT_M1/R_	0.6718	0.1073	0.5282	0.0763	0.5834	0.1016	3.66	0.016	1.64
FOOT_M1/L_	0.6344	0.0681	0.6659	0.1410	0.6525	0.0868	−0.65	0.526	−0.29
FOOT_S1/R_	0.7197	0.1025	0.5864	0.0947	0.6599	0.1254	3.17	0.020	1.42
FOOT_S1/L_	0.7229	0.1740	0.6305	0.0847	0.6482	0.1215	1.63	0.222	0.73

*Note*. Differences were calculated between the two sports groups using unpaired *t*-tests (false discovery rate [FDR] corrected). Value within brackets represents degrees of freedom; *d*, effect size (*Cohen's d*); L, left hemisphere; *M*, mean; M1, primary motor cortex; *n*, number of subjects; *p*, error probability (two-tailed); R, right hemisphere; S1, primary somatosensory cortex; SD, standard deviation; *t*, *t*-value.

**Table 4:** Group comparisons of fractional anisotropy (FA) in the respective corticospinal tracts (CST).

CST	Ballet (*n* = 10)		Handball (*n* = 12)		Controls (*n* = 22)		*t *(20)	*p*	*d*
	*M*	SD	*M*	SD	*M*	SD			
HAND_M1/R_	0.4372	0.0264	0.4305	0.0206	0.4298	0.0275	0.66	0.516	0.30
HAND_M1/L_	0.4144	0.0287	0.4456	0.0210	0.4304	0.0256	−2.94	0.016	−1.32
HAND_S1/R_	0.4058	0.0238	0.4192	0.0365	0.4139	0.0423	−1.00	0.377	−0.45
HAND_S1/L_	0.3794	0.0237	0.4192	0.0206	0.4057	0.0252	−4.22	0.001	−1.89
FOOT_M1/R_	0.4252	0.0198	0.4408	0.0269	0.4301	0.0297	−1.52	0.230	−0.68
FOOT_M1/L_	0.3834	0.0186	0.4671	0.0222	0.4302	0.0386	−9.45	6.48*E* ^−8^	−4.25
FOOT_S1/R_	0.3854	0.0304	0.4010	0.0234	0.4035	0.0239	−1.36	0.253	−0.61
FOOT_S1/L_	0.3586	0.0286	0.4561	0.0242	0.4132	0.0395	−8.68	1.28*E* ^−7^	−3.89

*Note*. Differences were calculated between the two sports groups using unpaired *t*-tests (false discovery rate [FDR] corrected). Value within brackets represents degrees of freedom; *d*, effect size (*Cohen's d*); L, left hemisphere; *M*, mean; M1, primary motor cortex; *n*, number of subjects; *p*, error probability (two-tailed); R, right hemisphere; S1, primary somatosensory cortex; SD, standard deviation; *t*, *t*-value.

**Table 5:** Group comparisons of radial diffusivity (RD) in the respective corticospinal tracts (CST).

CST	Ballet (*n* = 10)		Handball (*n* = 12)		Controls (*n* = 22)		*t* (20)	*p*	*d*
	*M*	SD	*M*	SD	*M*	SD			
HAND_M1/R_	0.00056	0.00004	0.00060	0.00002	0.00060	0.00005	−3.01	0.014	−1.37
HAND_M1/L_	0.00058	0.00004	0.00060	0.00003	0.00060	0.00040	−1.30	0.280	−0.60
HAND_S1/R_	0.00060	0.00004	0.00058	0.00004	0.00059	0.00007	1.63	0.190	0.52
HAND_S1/L_	0.00062	0.00003	0.00064	0.00003	0.00063	0.00005	−0.97	0.395	−0.70
FOOT_M1/R_	0.00061	0.00004	0.00057	0.00003	0.00060	0.00005	3.00	0.019	1.20
FOOT_M1/L_	0.00063	0.00004	0.00055	0.00003	0.00061	0.00005	6.30	1.60*E* ^−5^	2.41
FOOT_S1/R_	0.00064	0.00004	0.00063	0.00003	0.00065	0.00003	0.63	0.539	0.30
FOOT_S1/L_	0.00067	0.00003	0.00057	0.00003	0.00063	0.00005	7.19	4.63*E* ^−6^	3.50

*Note*. Differences were calculated between the two sports groups using unpaired *t*-tests (false discovery rate [FDR] corrected). Value within brackets represents degrees of freedom; *d*, effect size (*Cohen's d*); L, left hemisphere; *M*, mean; M1, primary motor cortex; *n*, number of subjects; *p*, error probability (two-tailed); R, right hemisphere; S1, primary somatosensory cortex; SD, standard deviation; *t*, *t*-value.
